# Antibiotic Resistance Results of *Helicobacter pylori* in a University Hospital: Comparison of the Hybridization Test and Real-Time Polymerase Chain Reaction

**DOI:** 10.1155/2020/8853298

**Published:** 2020-11-04

**Authors:** Zehra Kipritci, Yesim Gurol, Gulden Celik

**Affiliations:** ^1^Yeditepe University Hospital, İstanbul, Turkey; ^2^Acıbadem University School of Medicine, Department of Clinical Microbiology, Istanbul, Turkey; ^3^Bahçeşehir University, Department of Clinical Microbiology, Istanbul, Turkey

## Abstract

**Aim:**

*H. pylori* is a bacterial pathogen in the human stomach which infects about 50% of the world population. Untreated infection can lead to various diseases leading to cancer. Some of the *H. pylori* strains are asymptomatic, but some of them cause more severe diseases. Standard treatment protocol used for the treatment of *H. pylori* infection is triple therapy, which includes omeprazole as a proton pump inhibitor (PPI) and two antibiotics usually consist of amoxicillin and clarithromycin or metronidazole. In the recent years, because of the increase in the rate of antibiotic resistance, the eradication rate has decreased.

**Materials and Methods:**

We evaluated 140 patients who applied to a university hospital gastroenterology department and underwent biopsy during endoscopy. In these patients, we analysed floroquinolone and clarithromycin resistance using the GenoType® HelicoDR (Hain Life Science, Germany). We also used the real-time method for clarithromycin resistance.

**Results:**

We found the number and rate of floroquinolone resistance as 20 (25.6%) and clarithromycine resistance as 31 (39.7%). With the real-time PCR method, we detected clarithromycine resistance in 26 (33.3%) patients. These results were not statistically significant. *Discussion and Conclusion*. Our results show similarity to the other studies held in our country. There should be more studies for the policy of eradication through our country.

## 1. Introduction

*H. pylori* is a bacterial pathogen in the human stomach, which infects about 50% of the world population. It is usually transmitted in childhood, and in the absence of antibiotic treatment, life-long colonization occurs [1, 2]. Infection by *H. pylori* causes gastritis initially and, if allowed to persist, can induce a range of pathologies such as peptic ulcers, atrophic gastritis, intestinal metaplasia, and gastric cancer. *H. pylori* colonization of the human stomach usually occurs in childhood, but most of *H. pylori*-induced diseases occur in adulthood and persist in the oldest people, whereas none of these diseases develop in the majority of people infected with *H. pylori* (>80%) and they are asymptomatic throughout their life. The inflammatory response to infection varies due to genetic predisposition to disease and environmental factors [2, 3].

The basis of *H. pylori* treatment is antibiotics. The eradication rates are low in children and the reinfection is frequent. The successful eradication treatment regimen has not yet been established despite the use of various treatment protocols. Colonization under the mucus layer reduces the direct effect of antibiotic. Stomach acid reduces the antibiotic effect, and bacteria easily form resistance to antibiotics; this may be the reason of treatment failures [4, 5].

For eradication and triple treatment of a proton pump inhibitor (PPI) or ranitidine bismuth and two antibiotics, most frequently, amoxicillin and clarithromycin are usually used [6]. The duration of triple treatment is still controversial and varies between 7 and 14 days, depending on the centers [4]. The most common used antibiotics are macrolides (clarithromycin or azithromycin), imidazole (metronidazole or tinidazol), amoxicillin, and tetracycline [6]. The most prescribed antibiotic against *H. pylori* used as a part of the triple therapy is clarithromycin. According to consensus conferences, when the resistance rate reaches 20%, this antibiotic should not be used. Phase 3 trials in Europe have shown that when the prevalence of clarithromycin resistant is increased in *H. pylori* strains, the two antibiotics, PPI and bismuth, which are the four treatment modalities, should be the first line treatment method. Metronidazole can be used instead of clarithromycin because of resistance [6]. In the failure of *H. pylori* eradication, levofloxacin is used for the second drug, which is a fluoroquinolone group of antibiotics [7].

Although rapidly developing resistance problem is changing from country to country/region to region, *H. pylori* cannot be eradicated in 10–45% of triple therapy patients [4]. In developing countries, alternative antibiotic treatments are tested. These are four treatment, sequential treatment, and triple treatment which indicate other antibiotics such as levofloxacin, rifabutin, and furazolidane. Four treatment indicates bismuth, PPI, metronidazole, and tetracycline [6]. Antibiotic resistance of *H. pylori* is not regular in the world, low in developing countries, and low in developed countries consistent with prescribing frequency. For his reason, a sensitivity test is recommended before starting the treatment regimen [6]. Especially in developing countries such as Turkey, the detection of antibiotic resistance is important for the effectiveness of the treatment [7].

Due to the technical difficulties of culturing *H. pylori* and antibiograms, it is shown that easy and cheap methods are needed to determine resistance [8]. In the recent years, many molecular methods such as fluorescence in situ hybridization, PCR sequencing, real-time PCR, and in-line hybridization PCR have been developed as alternatives to conventional methods [9]. Clarithromycin resistance is determined by phenotypic and genotypic methods. Resistance detection with mutations such as A2142G, A2142C, A2143G, A2143G, and A2144G by molecular methods makes it easier and more accurate to obtain results [10]. These mutations are sought by PCR-RFLP using restriction endonucleases. Fluoroquinolone resistance is associated with mutations in 87 and 91 positions in the gyrA gene and has been shown to be detected by molecular methods [11].

Antibiotic resistance rates vary from region to region. Turkey also observed different rates of resistance in different regions. Our aim is to determine the resistance to clarithromycin and fluoroquinolones, which are the most commonly used antibiotics for *H. pylori* eradication, by the hybridization method which is a molecular method and to compare with the real-time PCR method.

## 2. Materials and Methods

The study was carried out approved with the ethical approval of the Yeditepe University Clinical Research Ethics Committee (decision no: 51/445). Gastric biopsy specimens taken during endoscopy from randomly selected 140 patients who were referred to the Gastroenterology Department of a University Hospital were placed in Nucliswab® (Salubris, USA) and delivered to the Clinical Microbiology laboratory. First DNAs obtained by DNA extraction were stored at −20°C. Gastric biopsy specimens first were crushed, and DNA isolation was carried out by using the QIAamp DNA Mini Kit (Qiagen, Hilden, Germany) extraction kit with the protocol recommended for the tissues.

### 2.1. Hybridization with the Genotype® HelicoDR (Hain Life Science, Germany) Kit

**Figure 1:**
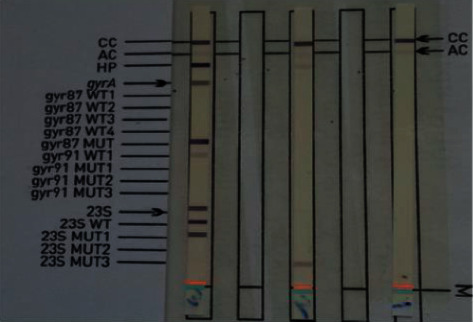
Patients who were *H. pylori* positive and negative with the Genotype® HelicoDR (Hain Life Science, Germany) kit. In the left patient HP band seen, the banding in the gyr87 MUT region is indicating a mutation in the gyrA region, that is, it is resistant to fluoroquinolone; at the same time, the region of 23S with the WT in the MUT1 band is seen in the band, i.e., we can say that *H. pylori* strains isolated from this disease are resistant to clarithromycin. In the patient in the middle, the HP band can be seen but gyrA and 23S bands cannot be seen. We can say that this patient is *H. pylori* positive. In the right patient, there is no band except for the control and amplification band, meaning that the patient has been evaluated as *H. pylori* negative.

In the isolated DNAs, the presence of *H. pylori* and the presence of fluoroquinolone and clarithromycin resistance was investigated using the Genotype® HelicoDR (Hain Life Science, Germany) kit (Figure 1).

### 2.2. Detection of Clarithromycin Resistance by Real-time PCR

In order to demonstrate the clarithromycin resistance in DNA, a real-time PCR-based PCR hybridization test was performed to detect point mutations. A fragment of 267 bp of the 23S rRNA gene of *H. Pylori* was multiplied by using HPYS (5′-TATGGTACCCGCATGATATTCCCATTAGCAGT-3′) and HPYA (5′-TAAGAGCTCAGGTTAAGAGGATGCGTCAGT-C-3′) primers. The amplified product was detected with two probes: the sensor probe, 5′ labeled with LC-Red 640 and 3′ phosphorylated (5′-GGCAAGACGGAAAGACC-3′; nucleotides 2504 to 2520), which hybridized with the region containing the mutation sites, and the anchor probe, which hybridized three bases upstream from the former and was 3_ labeled with fluorescein (5′-TGTAGTGGAGGTGAAA-ATTCCTCCTACCC-3′; nucleotides 2473 to 2501). Primers and probes were obtained from Sentromer DNA Technologies Ltd.

PCR and hybridization reactions were performed using a LightCycler thermocycler (Roche Diagnostics, Neuilly sur Seine, France). In the tubes of glass capillaries, 1, 6 *µ*l MgCl_2_ (25 mM), 0,4 *µ*l forward and reverse primers (eachone 20 *µ*M), 0,2 *µ*l anchor and sensor probes (eachone 20 *µ*M), and 2 *µ*l FastStart DNA Master Hybridization probes (Roche diagnostics) and indicate 3 *µ*l DNA, and PCR mixture was prepared to have a total volume of 20 *µ*l. The amplification program is as follows: In 95°C 10 minute (1 cycle), (senaturation), in 95°C 0 second, in 60°C 10 second, in 72°C 17 second (50 cycle), in 95°C 0 second (1 cycle), (melting), in 5°C 30 second (1 cycle) (cooling), and slow progress with a fluorescence drop of 0.1ºC in 85°C. After the procedure was completed, the *T*_*m*_ points were compared with the LightCycler instrument to evaluate.

Four different *T*_*m*_ points are seen in the study. It is expected that, for the wild type strain, A2142C, A2142G, and A2143G mutant strains are detected as at approximately 61.5, 58.0, 53.0, and 53.6 [12].

## 3. Results

The study included 140 biopsy specimens taken from the Yeditepe University Gastroenterology Department. Patients' ages ranged from 19 to 73 years, and patients were randomly selected.

Point mutations in the gyr87 and gyr91 gene regions were examined for the detection of resistance to fluoroquinolone antibiotics by using the Genotype® HelicoDR (Hain Life Science, Germany) kit. In this study, 78 patients were identified as positive. In these, 14 were found to be mutated in the gyr87 region, 6 in the gyr91 region, and 2 in both gene regions. A total of 20 (25.6%) patients had mutation in the gyrA gene region, namely, fluoroquinolone resistance. Point mutations in the A2146G, A2146C, and A2147G gene regions were examined for the detection of clarithromycin resistance using the Genotype® Helico DR (Hain Life Science, Germany) kit. Thirty-one (39.7%) of 78 patients were found to have clarithromycin resistance. 38.7% of them has A2142G, 67.7% of them has A2143G mutation, and none has A2142C mutation. Resistance to both fluoroquinolone and clarithromycin antibiotics was found in 11 (14.1%) patients. The resistance of clarithromycin was determined by the real-time PCR method in point mutations of A2142G, A2142C, and A2143G gene regions in 78 patients who were detected *H. pylori* positive by the hybridization method. Twenty-six (33.3%) patients were found to have clarithromycin resistance. 14.8% of them has A2142G, 85.2% of them has A2143G, and A2142C mutation is not detected. Comparing the results of hybridization and real-time PCR methods with clarithromycin resistance tests, mutation was detected in both methods in 24 (30.8%) patients. Comparisons of these two tests were not statistically significant when *p* value >0.05. It is accepted that the prekit is more accurate than the previous studies (Table 1).

## 4. Discussion and Conclusions

The standard treatment protocol for the eradication of *H. pylori* is triple therapy. It consists of a proton pump inhibitor (PPI) such as omeprazole and two antibiotics, usually amoxicillin, clarithromycin, or metranidazole. Studies have shown that this treatment eradicated approximately 80% of *H. pylori* at the beginning of the 1990s. But, later, the eradication success rate dropped below 60%. This is related to the increase of clarithromycin and metranidazole resistance in the world. Fluoroquinolones, such as levofloxacin and moxifloxacin, are often used as second-line treatment rescuers [11]. Antibiotic susceptibility testing should be performed according to the 5th Maastricht guideline published in 2017 if the second generation treatment fails [13].

Treatment failures are related to the age of the patient, cigarette use, bacterial burden before the treatment, genotype of the bacterium, and drug compliance of the patient. However, most of the treatment failures are due to resistance to the first choice antibiotics [7]. The use of similar antibiotics in different diseases causes to develop resistance to antibiotics. Sensitivity testing is recommended for antibiotics that can be used for increased resistance to antibiotics. Phenotypic methods for susceptibility testing are difficult because *H. pylori* is a slow-breeding bacterium under optimal culture conditions. Because the antibiotic resistance in this microorganism depends on the point mutation, it is more convenient to use genotypic methods as an alternative. Since these methods can be applied to direct biopsy material and stool specimens, it is an important step in getting faster results [6].

Clarithromycin is the most preferred antibiotic in *H. pylori* eradication, but the rate of eradication has decreased from 88% to 20% due to clarithromycin resistance. However, clarithromycin is still the first treatment option in the world [7, 11]. Clarithromycin resistance is seen at different rates in different regions. The studies showed that clarithromycin resistance in Europe has increased from 9% in 1998 to 17.6% in 2008. In Japan, the rate of resistance, which was 7% in 2000, was 27.7% in 2006 [14]. According to studies conducted in France, the resistance of clarithromycin increased from 14.3% in 1997 to 22.2% in 2014. According to the 5 Maastricht guidelines, when the resistance of clarithromycin is increased to over 20%, second-line treatment should be applied by removing clarithromycin from the treatment [15]. The clarithromycin resistance in Turkey is between 16.4% and 48.2%. The resistance of clarithromycin was found to be 40.5% in 2008 by Sezgin et al. in Mersin [16]. In the study conducted in the Çukurova region in 2011, the resistance of clarithromycin was found to be 7.7% by Yula et al. [17], 36.7% in the year 2013 [7], and Maçin et al. found 30.1% in Ankara in 2015 [18].

In our study, clarithromycin resistance was found to be 39.7% with the Genotype® HelicoDR (Hain Life Science, Germany) kit and 33.3% with the real-time PCR method. These results demonstrate familiarity with the average resistance rate detected in Turkey with other studies.

The Genotype® HelicoDR (Hain Life Science, Germany) kit is used as a hybridization kit; Cambau et al. (2006–2009) conducted studies in France to compare the clarithromycin and fluoroquinolone resistance rates with MIC (E-test) methods. In this study, the sensitivity and specificity rates were 94 and 98% in the clarithromycin resistance test and 87 and 98.5% in the levofloxacin resistance test, respectively [11].

Levofloxacin is a fluoroquinolone whose activity against *H. pylori* is important. It is used as a secondary drug in failure of *H. pylori* eradication [19]. However, the number of studies on levofloxacin resistance is less [20]. According to one study, eradication rate is 80.8% with clarithromycin over 96% when levofloxacin was used [19]. In the study performed by Caliskan et al., the rate of resistance to levofloxacin was 29.5% [7]. Çağdaş et al. found the rate of levofloxacin resistance as 18.2% [21]. In Brazil, Martins and colleagues found 11.1% [22]. In Pakistan, the rate of levofloxacin resistance was 62.3% in the study conducted by Rajper et al. [23]. In this study, which we conducted with the same method, the rate of fluoroquinolone resistance was found to be 25.6%. This rate is generally correlated well with the rate of fluoroquinolone resistance in Turkey (Table 2).

The resistance rates are increasing worldwide. For this reason, susceptibility testing should be performed before treatment, but molecular methods are preferred to obtain faster and more accurate results than *H. pylori* infection. Molecular methods are also expensive and seem difficult to use on a routine basis. The European Helicobacter and microbiota working group (EHMSG) and also in Turkey, the *H. pylori* working group of Gastroenterological Association support make more and larger studies on these bacteria. The guides such as the Maastricht V/Florence Consensus Report by EHMSG contains recent information about *H. pylori*. For update, especially for the eradication, there should be more studies conducted in this field.

## Figures and Tables

**Table 1:** Antibiotic resistance rates of *H. pylori*-positive patients.

	Genotype® Helico DR
Fluoroquinolone resistance	20 (25.6%)
Clarithromycin resistance^*∗*^	31 (39.7%)
Fluoroquinolone and clarithromycin resistance	11 (14.1%)

^*∗*^Clarithromycin resistance was detected in 26 (33.3%) patients also by the real-time PCR method.

**Table 2:** Drug resistance of *H. pylori* in Turkey.

Year/region	Method	KLA (%)	LEV (%)	MTZ (%)	TE (%)	AMO (%)	References
2003/Ankara (*n* = 87)	PCR-RFLP	27.6	—	—	—	—	Bağlan et al. [24]
2007/İzmir (*n* = 110)	RT PCR	48.2	—	—	—	—	Önder et al. [25]
2008/Mersin (*n* = 37)	PCR-RFLP	40.5	—	—	—	—	Sezgin et al. [16]
2011/Çukurova (*n* = 79)	PCR- RFLP/Agar dilusion	7.7/8.9	—	—	—	—	Yula et al. [17]
2013/Mersin (*n* = 94/11)	PCR-RFLP/E-test	18.1/18.2	—	45.5 (E-test)	9.1 (E-test)	0	Çağdaş et al. [21]
2015/İstanbul (*n* = 98)	E-test	36.7	29.5	35.5	0	0	Çalışkan et al. [7]
2015/Ankara (*n* = 93)	E-test	30.1	—	48.4	0	0	Maçin et al. [18]
2016/İstanbul (*n* = 78)	RT PCR/Helico DR	33.3/39.7	25.6	—	—	—	Kipritçi et al. (this article)

CLA: clarithromycin, LEV: levofloxacin, MTZ: metronidazole, TE: tetracycline, AMO: amoxicillin, *n*: total number of patients worked.

## Data Availability

The data used to support the findings of this study are included within the article.
